# Compartment Syndrome Secondary to Calcium Gluconate Extravasation

**DOI:** 10.7759/cureus.42237

**Published:** 2023-07-21

**Authors:** Derek S Weimer, Sydney Jones, Tanya Ramadoss, Una Milovanovic, Mohammadali M Shoja, Gary Schwartz

**Affiliations:** 1 Department of Medical Education, Dr. Kiran C. Patel College of Allopathic Medicine, Nova Southeastern University, Fort Lauderdale, USA

**Keywords:** limb ischemia, extravasation injury, major limb amputation, calcium gluconate, hand compartment syndrome

## Abstract

This case report highlights a rare yet severe complication of calcium gluconate extravasation, namely, compartment syndrome. We present the case of an 86-year-old female who developed compartment syndrome following an extravasation of intravenously administered calcium gluconate for the management of hyperkalemia. Initially, mild erythema and edema were observed at the site of extravasation, which eventually progressed to severe pain, a reduction in the joint range of motion due to increased compartment pressure. Despite undergoing a series of fasciotomies, the patient's condition did not improve, and extensive tissue necrosis and gangrene necessitated amputation. This case emphasizes that calcium gluconate extravasation can lead to life-threatening complications, such as compartment syndrome, underscoring the critical importance of employing proper infusion techniques.

## Introduction

Intravenous calcium gluconate is commonly utilized for the management of various conditions, such as cardiac arrest, cardiotoxicity associated with hyperkalemia or hypermagnesemia, and hypocalcemia in premature neonates [1,2]. However, the extravasation of calcium-containing peripheral solutions into the surrounding soft tissues can result in rapid and significant tissue edema, induration, local erythema, and tenderness [3]. In severe cases, this can lead to local vasoconstriction and tissue necrosis [1]. Unusual reactions that have been reported following calcium gluconate extravasation include calcinosis cutis [2], bullous skin reactions [4], cellulitis [5], and transient radial nerve damage resulting in wrist drop [6]. Compartment syndrome is a rare yet serious complication that can occur as a result of calcium gluconate extravasation, which has been previously documented in neonates [2]. Acute compartment syndrome arises from a combination of decreased intracompartmental space and/or increased fluid volume within the compartment, along with decreased compliance of the surrounding fascia [7]. This leads to an elevation in compartment pressure, subsequently raising venous capillary pressure and impeding venous outflow. If the intracompartmental pressure surpasses arterial pressure, arterial inflow becomes compromised. The resulting blood flow stasis leads to ischemia and, in later stages, irreversible tissue necrosis [7].

Clinical features commonly associated with compartment syndrome are recognized as the "5Ps": pain out of proportion, pallor, paresthesia, paralysis, and pulselessness [8]. Although compartment syndrome is primarily linked to trauma, it can also occur due to nontraumatic and iatrogenic causes. Iatrogenic causes encompass factors, such as anticoagulation therapy, the use of pressurized infusion pumps, and, importantly, extravasation injuries [9]. In this case, we present the instance of an 86-year-old patient who developed compartment syndrome following the administration of intravenous calcium gluconate. The objective of the present report is to enhance awareness about the potential hazard of compartment syndrome, a severe and life-threatening complication associated with the administration of calcium gluconate.

## Case presentation

An 86-year-old female presented at the emergency department complaining of extreme weakness. Her medical history included hypertension managed with spironolactone and verapamil and anemia managed with iron supplementation. An electrocardiogram (EKG) revealed bradycardia with a heart rate in the 40s, and her serum potassium level was critically elevated at 6.5 mmol/L. Two doses of intravenous calcium gluconate (1 gram in 50 mL each) were administered one hour apart through the right dorsal wrist/forearm to address hyperkalemia. In addition, treatments, such as sodium bicarbonate, insulin, albuterol, and Kayexalate, were administered. Subsequently, it was determined that the likely cause of the hyperkalemia was the treatment with spironolactone. Consequently, the patient was admitted to the hospital for further evaluation and management.

**Figure 1:**
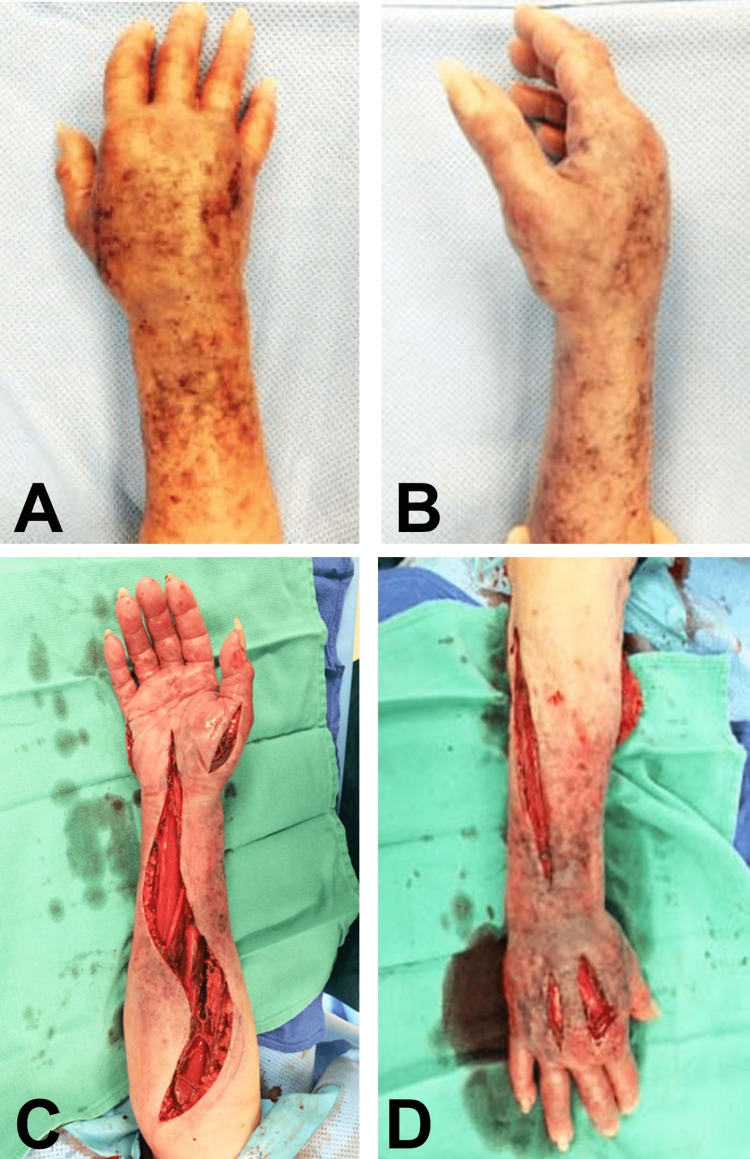
Pre-operative and initial intra-operative photographs. Pre-operative anterior (A) and lateral view (B) of the right hand, demonstrating edema and erythema after calcium gluconate extravasation onto the dorsal aspect of the hand. Intra-operative photographs of the volar (C) and the dorsal (D) incisions at the time of fasciotomy.

At the time of administering intravenous calcium gluconate, a small amount of local ecchymosis was observed, but it was considered insignificant at the time. However, over the next two days, mild erythema and edema were noted in the right hand, wrist, and forearm. To address these symptoms, warm compresses were applied to the site, and the right upper extremity was elevated (Figures 1A, 1B). On the second day, extravasation was suspected when the patient presented with increased edema and ecchymoses over the injection site on the dorsum of the right hand. Upon evaluation, the right hand exhibited diffuse ecchymosis, mottling, limited range of motion, and some tenderness. The sensation was intact, and capillary refill was less than three seconds. Given the normal sensory examination and the absence of pain during passive extension of the digits, the patient was closely monitored, and no further intervention was deemed necessary.

**Figure 2:**
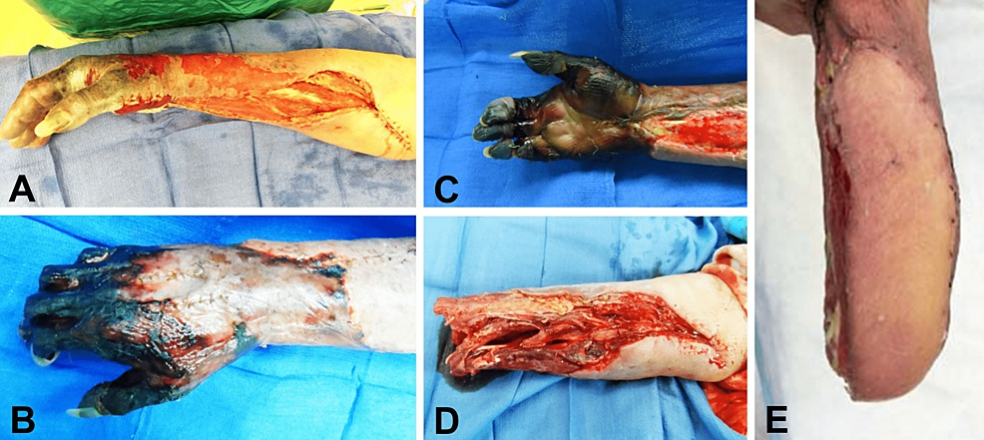
Intra-operative photographs on the 19th day. Full-thickness necrosis demarcating at the mid-forearm level (A), and gangrenous changes of the right hand and distal forearm (B and C). Intra-operative views of the forearm before (D) and after (E) application of the tensor fascia lata free-flap.

The symptoms continued to worsen, with the patient experiencing severe pain in the hand and forearm, particularly during passive extension of the fingers. Compartment pressure measurements revealed an increase in both the dorsal and volar compartments of the forearm. On the fourth day of hospitalization, the patient underwent a right hand and forearm fasciotomy (Figures 1C, 1D). Two weeks after the initial fasciotomy, the right hand and forearm exhibited full-thickness necrosis, which was demarcated at the mid-forearm level. In addition, gangrenous changes were observed in the hand and distal forearm (Figures 2A, 2B, 2C). Furthermore, there was a complete loss of motor and sensory function in the right hand. Subsequent explorations and debridement procedures were performed, leading to an amputation at the mid-distal forearm on the 19th day. Following the amputation, a tensor fascia lata free-flap coverage procedure was carried out (Figures 2D, 2E). Unfortunately, the patient's condition deteriorated due to comorbidities. Complications included severe anemia, respiratory failure necessitating intubation, deep vein thrombosis in the left lower extremity, atrial fibrillation, and pneumonia, ultimately resulting in the patient's demise.

## Discussion

Extravasation refers to the leakage of an injected substance from the blood vessels, leading to its flow into the surrounding tissues [10]. Symptoms associated with extravasation can manifest immediately or develop gradually over the course of several days or weeks. Common clinical findings include pain, a burning sensation, and edema around the site of injection. In the case of our 86-year-old female patient, small ecchymoses were observed during the administration of calcium gluconate, but they were initially considered insignificant. However, once extravasation is suspected, the first crucial step is to immediately discontinue the infusion [10,11]. Extravasation is particularly problematic when it involves vesicant substances, including calcium gluconate [12]. Calcium gluconate is the calcium salt of gluconic acid and is typically administered intravenously and is preferred over calcium chloride due to its lower risk of tissue necrosis if extravasation occurs [1]. However, despite this lower risk profile, calcium gluconate extravasation can still give rise to life-threatening complications, such as compartment syndrome. In the case described here, compartment syndrome developed four days after administration of calcium gluconate for the management of hyperkalemia.

Compartment syndrome arises from an increase in interstitial fluid pressure within the fibro-osseous space, resulting in compromised blood flow. Significant reduction in blood flow leads to ischemic damage to muscular and neurovascular structures. Diagnosis is primarily based on the patient's clinical history and physical examination. Key indicators include pain, pallor, paresthesia, paralysis, and pulselessness [8,13]. In the case of our patient, she presented two days after receiving the intravenous infusion with edema at the intravenous injection site and a small area of ecchymosis on the dorsum of her right hand. Over time, the effects of extravasation progressively worsened, eventually culminating in the development of compartment syndrome four days after the infusion.

The pathophysiology of compartment syndrome secondary to calcium gluconate extravasation is complex and involves multiple factors. The exact minimum volume and concentration of calcium gluconate required to trigger compartment syndrome has not been established in previous studies. Calcium gluconate, being a hypertonic solution, creates an osmotic gradient when it extravasates, leading to the movement of fluid from the interstitial space into the extravascular compartment. This accumulation of fluid within the compartment increases the pressure, causing the non-compliant fascial layers to expand and compress the surrounding neurovascular structures [14]. In addition to the mechanical effects, calcium gluconate also triggers an inflammatory response in the surrounding tissue. This inflammatory response leads to vasodilation and increased capillary permeability, further contributing to the pressure within the fascial compartment [14,15,16]. As the condition progresses, nerve function can become compromised, resulting in symptoms, such as paresthesia and paralysis [13]. Overall, the elevated compartment pressure hampers microcirculation and leads to tissue ischemia, ultimately resulting in tissue necrosis.

The confirmation of compartment syndrome can be achieved by measuring intracompartmental pressure. If the compartment pressure exceeds 30 mmHg above the diastolic pressure, it is an indication for performing a fasciotomy [15]. Fasciotomies are surgical procedures performed to alleviate high compartment pressure, allowing for continued edema while preventing further neurovascular damage. In hand fasciotomies, the release typically includes the carpal tunnel and the 10 intrinsic compartments of the hand, including the adductor, four dorsal interossei, hypothenar, thenar, and three volar interossei compartments [13]. In the case described above, a series of fasciotomies were performed to address the elevated compartment pressures. However, despite undergoing debridement procedures, the patient experienced extensive tissue necrosis and gangrene, which did not improve. It was determined that no medical intervention could restore viability to the affected arm, necessitating amputation as the final course of action. The progressive nature of tissue necrosis may have been partially attributed to the local tissue calcium salts deposits, as well as the increased compartment pressure. In addition, the patient's underlying systemic disorders, such as anemia, might have also contributed to the progression and worsening of compartment syndrome.

## Conclusions

Calcium gluconate extravasation can give rise to severe complications, such as compartment syndrome characterized by increased pressure within the fibro-osseous space and compromised tissue perfusion. The exact minimum volume of calcium gluconate required to trigger compartment syndrome remains uncertain. In confirming the diagnosis, measuring intracompartmental pressure is essential, and fasciotomies are performed to relieve the elevated compartment pressure. In the case of the patient described here, a series of fasciotomies and debridement procedures were carried out; however, extensive tissue necrosis and gangrene ultimately necessitated amputation. Recognizing the potential risk of compartment syndrome as a life-threatening complication associated with calcium gluconate extravasation is of utmost importance. It is critical to adhere to proper infusion techniques to prevent complications arising from calcium gluconate extravasation.
